# Intensive care nurses' knowledge of enteral nutrition at public hospitals in Sana'a, Yemen: a cross-sectional survey

**DOI:** 10.12688/f1000research.25041.1

**Published:** 2020-07-22

**Authors:** Talal Ali Hussein Al-Qalah, Gamil Ghaleb Alrubaiee

**Affiliations:** 1 Department of Applied Medical Sciences, Al-Razi University, Sana'a, Al-Amanah, 00967, Yemen; 2 Department of Community Health, Al-Razi University, Sana'a, Al-Amanah, 00967, Yemen

**Keywords:** Enteral nutrition, tube feeding, nurses’ knowledge, intensive care units, public hospitals, Yemen

## Abstract

**Background:** Nurses have a pivotal role in initiating and managing enteral nutrition (EN) and monitoring any potential complications. Yet, it is unclear whether Yemeni nurses have adequate knowledge to deliver and manage enteral nutrition safely. Therefore, the aim of this study was to assess the level of ICU nurses’ knowledge regarding the management of EN.

**Methods:** A descriptive cross-sectional study was conducted from February 2019 to March 2019. A probability sampling method was used to recruit 174 nurses from four public hospitals in the capital city of Yemen. A self-administered 17-item questionnaire related to ICU nurses' knowledge regarding EN intervention was used to collect the data.

**Results:** Out of the 174 respondents, 60.9% were females, 48.9% were aged between 20 and 25 years and 66.1% had a 3-year nursing diploma. Most respondents (79.3%) had one to five years of working experience as nurses, while almost two-thirds (70.7%) had one to five years of working experience as an ICU nurse. Above half (59.2%) of the respondents had never attended training courses on EN management and 65.5% specified that the college or institute of nursing was the source for their knowledge about EN management. Only 10.9% of the respondents had an adequate level of knowledge, while (43.1%) of them had a moderate level of knowledge and 46.0% of them had an inadequate level of knowledge regarding the EN management. Significant associations between the level of ICU nurses' knowledge of EN management and their level of education and the sources of knowledge were detected.

**Conclusions:** The significant gap in ICU nurses’ knowledge regarding EN management identified implies the need to upgrade and refresh of the ICU nurses' knowledge by implementing a regular training program concerning EN management.

## Introduction

Critically ill patients need regular enteral nutrition (EN) as an essential intervention to fulfil the body’s dietary and physiological requirement ^1^. EN is the recommended method of nutritional support for ICU ill patients who need extra nutritional calories because of the increased metabolic rate ^2^. It is considered as a medical therapy in law; hence, it should not be initiated only after ethical considerations have been made.

Previously, delivering of EN to critically ill patients was considered as a type of supportive care and not a therapeutic intervention ^3^. Based on its benefits, it is more than a supportive therapy. It has been found that EN protects critically ill patients from malnutrition and subsequently improves body immunity and healing of tissues, which decreases their physiological stress effect, peptic ulcer, rates of infection ^4, 5^, the inflammatory response and maintains the function and integrity of the bowel ^3, 6^. Although maintaining balanced feeding via EN improves patients' health-related-outcomes, overfeeding leads to an increase in the mortality rate and must be avoided ^7^.

Malnutrition among ICU ill patients is a universal public health concern, with a prevalence rate ranging from 40 to 60% ^8^. According to previous studies, almost 70% of ICU patients acquired malnutrition during hospitalization ^9^. To overcome this serious problem, EN should be initiated as early as possible for critically ill patients to avoid any risk of morbidity and mortality, which usually increases due to long patient hunger ^10^. Previous studies and nutritional guideline have recommended that EN should be started within 24 to 48 hours of patients' admission to the ICUs or after the stability of the hemodynamic state, specifically after cardiac resuscitation ^11^. In other words, once critically ill patients are admitted to ICUs, delivering appropriate nutrition becomes imperative ^12^.

Despite the benefits of EN to critically ill patients, this process is usually associated with many complications, particularly if performed without an adequate level of knowledge ^13, 14^. Inappropriate delivering and poor management of EN can result into several complications, such as tube blockage or mal-positioning, nausea, vomiting, pulmonary aspiration, overfeeding, diarrhoea, treatment-related complications and delivery-site related complications like infection and agitation ^15^. ICU nurses play a significant role in preventing such complications because they are responsible for identifying patients' nutritional risk, the calories needed, initiating and managing EN and monitoring any potential complications ^16, 17^. Accordingly, they must be knowledgeable enough to administer EN to critically ill patients safely ^14, 16^. Therefore, the aim of the present study was to explore Yemeni ICU nurses’ knowledge regarding management of EN. Specifically, our study aimed to answer the following two research questions:

1. What is the level of Yemeni ICU nurses’ knowledge regarding EN management?
2. Are there differences in the ICU nurses’ knowledge level regarding EN management based on their socio-demographic characteristics?

## Methods

### Design

A descriptive cross-sectional study design was used to assess the knowledge level of ICU nurses regarding EN management.

### Setting

The study was conducted from February to March 2019 in ICUs at four public hospitals in Sana'a, the capital city of Yemen. The four public hospitals are Al-Thawra Modern General Hospital, Al-Kuwait University Hospital, Al-Sabeen Maternity and Child Hospital, and the Republican Teaching Hospital Authority. These hospitals were selected as they are the referral hospitals for most people and the service fees are low.

### Sample size determination

According to Al-Hawaly, Ibrahim, and Qalawa results, 71.1% of the respondents had a satisfactory total level of knowledge about EN ^18^. Because our study was a cross-sectional, the following equation was used to calculate sample size: N=4pq/d ^2^, where; N: required sample size, p: expected proportion of sample (from previous studies), q = 100 – p, and d= wanted precision (10% was taken in this study). Accordingly, a sample of 163 nurses was calculated. Assuming attrition of 10% = 16 + 163 = 179 nurses was required.

### Sample and sampling method

Stratified sampling was utilized to select the participants from the above-mentioned public hospitals. A list of the ICU nurses’ name was obtained from each hospital. The required sample was drawn randomly from each list using a systematic random technique. All ICU nurses who had at least 6 months working experience, including both males and females with different educational qualifications, who were involved directly in ICU patients care, full-time employees and had agreed to participate were eligible to take part in this study. Nurses who included in the pilot study and those who were unwilling to participate were excluded. Based on these criteria, the eligible participants were approached by the researchers at their workplace. Out of 384 ICU nurses, 174 nurses were included in this study.

### Study instrument

Based on a comprehensive review of the previous related studies, a self-administered questionnaire was developed by the researchers. The questionnaire consists of 17 questions with four possible options to be answered. The final questionnaire (see *Extended data* ^19^ for a blank copy) is divided into two sections as follow:

The first section is related to the socio-demographic characteristics of the participants: age, sex, level of education, working experience as a nurse, working experience as ICU nurse, training courses on EN and sources of knowledge about EN.

The second section is related to knowledge of ICU nurses regarding EN management and is divided into three subsections:

1. Knowledge of ICU nurses regarding before EN administration, which includes eight questions with a total of 32 responses.

2. Knowledge of ICU nurses regarding during EN administration, which involves four questions with a total of 16 responses.

3. Knowledge of the ICU nurses regarding after EN administration, which involves five questions with a total of 20 responses.

### Scoring system

The 17 questions related to knowledge were assessed with “Yes” and “No” options. After correction of some reverse statements, a score of 1 was given for each correct response, while a score of 0 was given for each “incorrect” response. The maximum score for all correct answers was 68. Correct answers were calculated to obtain total scores for all questions of the three subsections. A score of 50% or less was considered inadequate, 51–75% moderate, while 76% and above was considered as adequate ^20^.

### Validity and reliability

Three experts in the EN from hospitals and Al-Razi University were invited to participate in examining the content validity for the instrument used in this study. Their comments concerning the tool accuracy, relevance, consistency, comprehensiveness and applicability for implementation were taken in consideration. A pilot study was conducted on 40 ICU nurses. Cronbach’s alpha test was performed to examine the reliability of the questionnaire items. The result of the alpha was 0.78, which is acceptable.

### Data collection

A self-administered questionnaire was distributed during the period of February to March 2019. The ICU nurses in the selected hospitals were invited to fill the questionnaire. Out of 179 questionnaires distributed, 174 were completed correctly and included in the final analysis.

### Data analysis

The participants' responses were entered, cleaned, checked and explored using statistical software (IBMSPSS), version 22.0. The analyzed data was described using the mean values and standard deviations for continuous variables as well as the frequency and percentages for the categorical variables. A multinomial logistic regression and Chi-square tests were conducted to find out the associations between the ICU nurses’ knowledge and the selected socio-demographic variables. A *p*-value of ≤0.05 was reported as statistically significant.

### Ethical consideration

Ethical clearance from the Ethics Committee of Al-Razi University was obtained for the current study. Then, an official written permission was also obtained from the managers of the selected hospitals prior to conducting the study. A written consent from all involved nurses was obtained prior to conducting the study.

## Results

### Participants’ socio-demographic characteristics

Almost half of the participants' age (48.9%) ranged from 20 to 25 years with a mean ±SD of 26.94±4.31. Most of them (60.9%) were females and had a 3-year nursing diploma (66.1%). Regarding their previous experience, 79.3% of the participants had 1 to 5 years of working experience as nurses, while 70.7% of them had working experience as ICU nurses for the same duration. More than half of the nurses (59.20%) had never attended training courses on EN, whereas 61.5% of them specified that colleges or institutes of nursing were a source for their knowledge about EN. Further details of socio-demographic characteristics of the participants are presented in Table 1. De-identified socio-demographic characteristics, in addition to individual-level responses to the questionnaire, are available as *Underlying data* ^21^.

**Table 1.** Participants’ socio-demographic characteristics (n=174).

Socio-demographic characteristics	Total n (%)
**Age (yrs)**	
20–25 years	85 (48.9)
26–30 years	60 (34.5)
31–35 years	20 (11.5)
≥36 years	09 (05.2)
**Sex**	
Male	68 (39.1)
Female	106 (60.9)
**Level of education**	
Diploma	115 (66.1)
Bachelor	55 (31.6)
Master	4 (2.3)
**Working experience as a nurse (yrs)**	
1–5	138 (79.3)
6–10	25 (14.4)
˃10	11 (6.3)
**Working experience in ICU (yrs)**	
1–5	123 (70.7)
6–10	32 (18.4)
˃10	19 (10.9)
**Training courses on enteral nutrition**	
Yes	71 (40.8)
No	103 (59.2)
**Sources of knowledge about enteral nutrition**	
College or Institute of Nursing	114 (65.5)
Consulting colleagues	23 (13.2)
In-service training courses	24 (13.8)
Others	13 (07.5)

### ICU nurses’ knowledge regarding EN management

**Figure 1.**
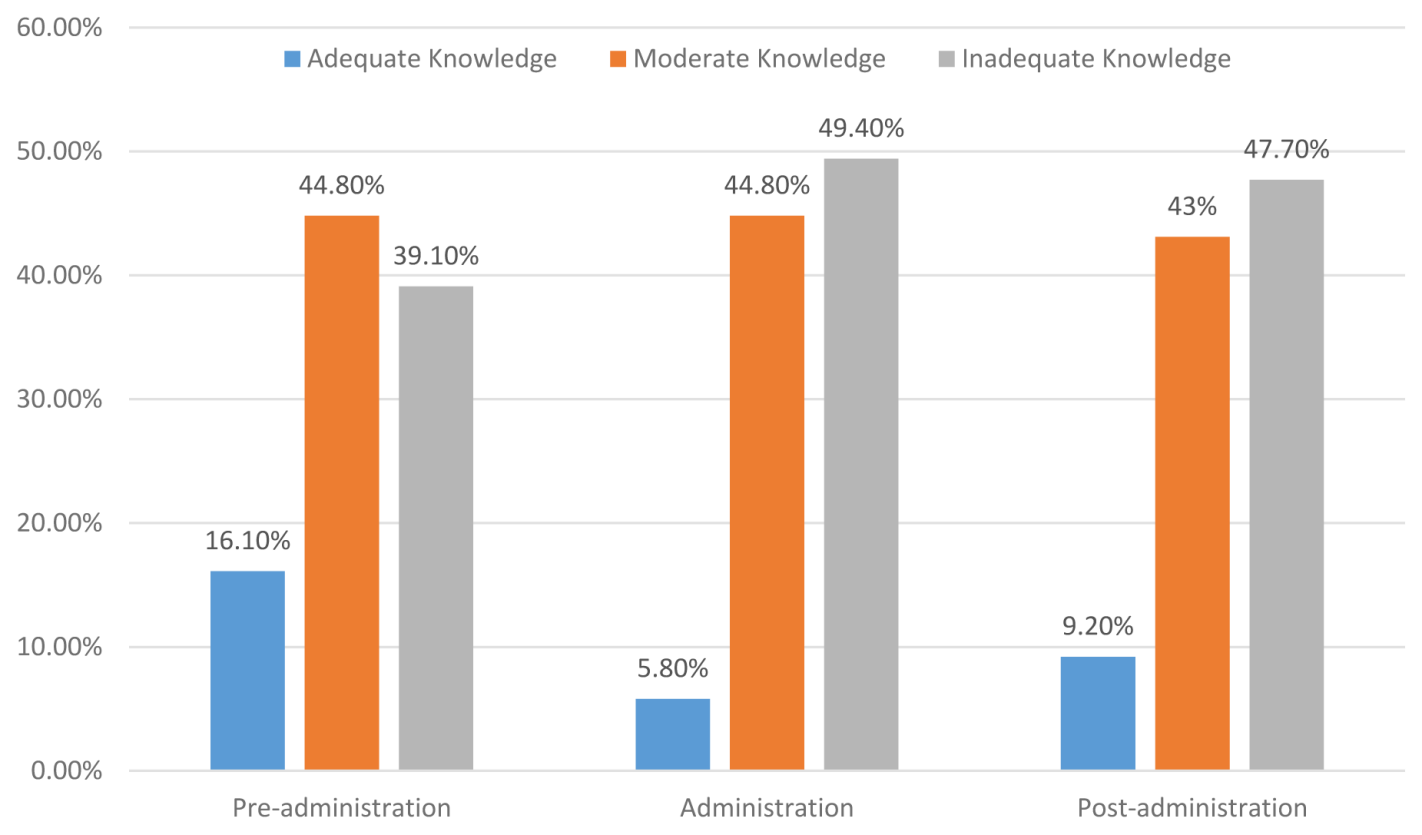
Intensive care nurses’ knowledge regarding EN management.

The results showed that only 16.1% of the participants had an adequate level of knowledge, while 44.80% of them had a moderate level of knowledge and 39.10% reported an inadequate level of knowledge about pre-administration of EN. Concerning the level of the participants’ knowledge on administration of EN, the results showed that the majority (49.40%) of them had an inadequate level of knowledge regarding administration of EN. However, 5.80% of the participants had an adequate level and 44.80% of them reported a moderate level of knowledge on administration of EN. Regarding the level of knowledge of EN following administration, the results revealed that most (47.70%) of the ICU nurses had an inadequate level of knowledge, and 9.20% had an adequate level, while 43% of them had a moderate level of knowledge about management following adminsitration of EN. The detailed results are demonstrated in Figure 1.

### Overall level of the ICU nurses’ knowledge regarding EN management

**Figure 2.**
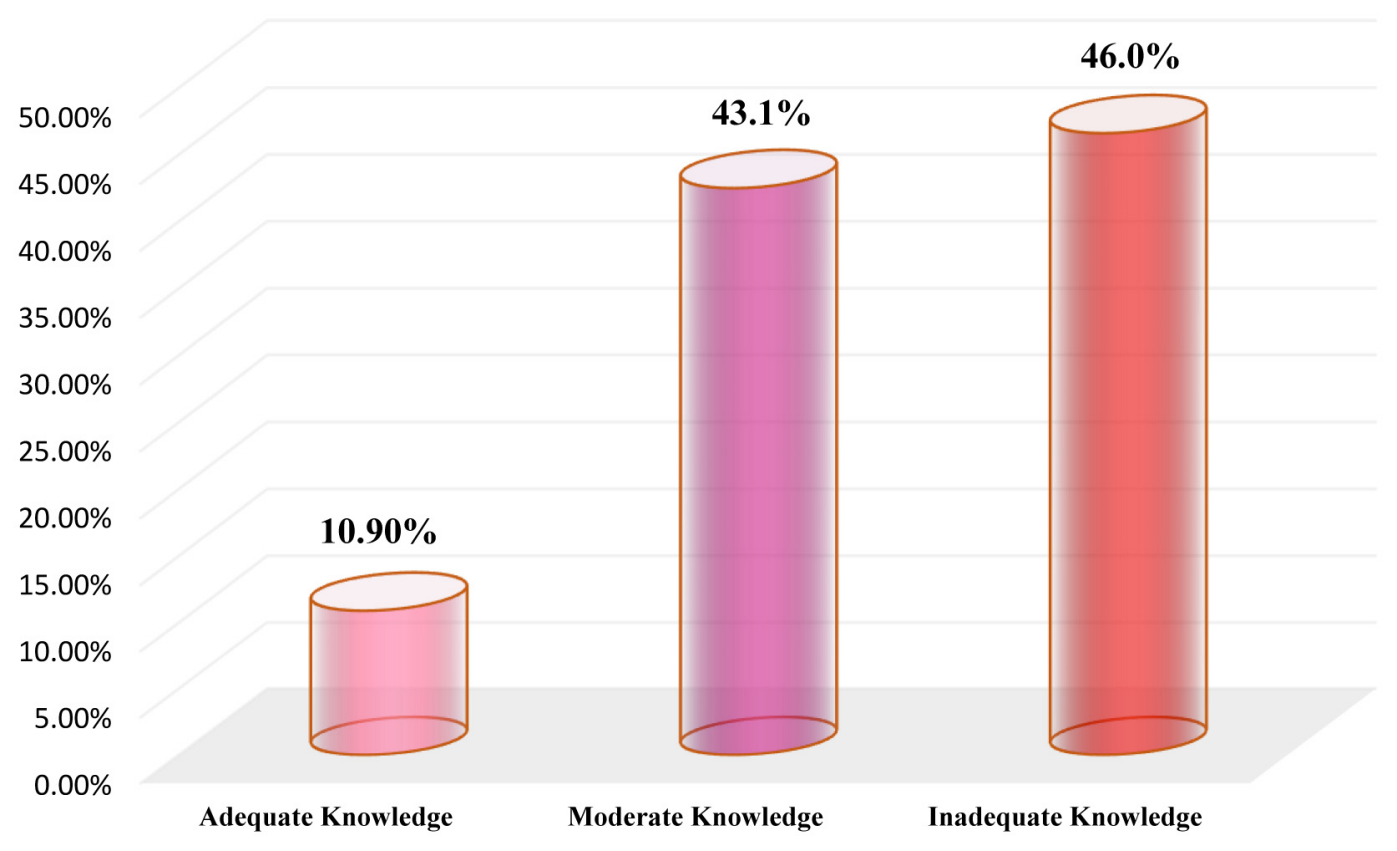
Overall level of the ICU nurses’ knowledge regarding EN management.

Concerning the overall level of ICU nurses’ knowledge on the different items of EN management, the results of the current study showed that the most (46.0%) of the participants had an overall inadequate level of knowledge and only (10.9%) had an overall adequate level of knowledge, while (43.1%) had an overall moderate level of knowledge regarding the EN management. The results are illustrated in Figure 2.

### Association between the ICU nurses’ characteristics and their knowledge level of EN

The current results indicated that there was a significant association between the level of education and sources of knowledge about EN and the overall level of participants’ knowledge of EN management ( *P*=0.011 and *P*=0.030, respectively). However, there were no significant associations between the participants’ age, sex, experience as nurses, experience as ICU nurses and training courses and the overall level of knowledge regarding EN management ( *P*= 0.276, 0.626, 0.425, 0.204 and 0.714, respectively). The detailed results of the association are presented in Table 2.

**Table 2.** Association between the ICU nurses’ characteristics and their knowledge level of EN.

Socio-demographic characteristics	Knowledge level			
	Adequate	Moderate	Inadequate	*P*-value
**Age (years)**				
20–25 years	7(8.3)	37 (43.5)	41(48.2)	0.276
26–30 years	6(10.0)	24(40.0)	30(50.0)	
31–35 years	5(25.0)	8(40.0)	7(35.0)	
≥36 years	1(11.1)	6 (66.1)	2(22.2)	
**Sex**				
Male	6(8.8)	32(47.1)	30(44.1)	0.626
Female	13(12.3)	43(40.6)	50(47.2)	
**Level of education**				
Diploma	8(7.0)	57(49.6)	50(43.5)	0.011 *
Bachelor	9(16.4)	17(30.9)	29(52.7)	
Master	2(50.0)	1(25.0)	1(25.0)	
**Working experience as a nurse (years)**				
1–5	12(8.7)	59(42.8)	67(48.6)	0.204
6–10	6 (24.0)	10(40.0)	9(36.0)	
˃10	1(9.1)	6(54.5)	4(36.4)	
**Working experience in ICU (years)**				
1–5	12 (9.8)	52(42.3)	59(48.0)	0.425
6–10	6(18.8)	15(46.9)	11(34.4)	
˃10	1(5.3)	8(42.1)	10(52.6)	
**Training courses on enteral nutrition**				
Yes	8(11.3)	28(39.4)	35(49.3)	0.714
No	11(10.7)	47(45.6)	45(43.7)	
**Sources of knowledge about enteral nutrition**				
College or Institute of Nursing	11(9.6)	56(49.1)	47(41.2)	0.030 *
Consulting colleagues	6(26.1)	8(34.8)	9(39.1)	
In-service training courses	2(8.3)	8(33.3)	14(58.3)	
Others	0(0.0)	3(23.1)	10(76.7)	

* *P-value* is significant at ≤ 0.05 level

## Discussion

The results of the current study revealed that the age of the majority of the ICU nurses’ was 20 years old and above. This result indicate that most participants were fresh graduates who were assigned together to work in ICUs. This finding is consistent with the result of a previous study ^16^ which reported that over half of participants’ age ranged between 26 and 35 years old. However, our result is inconsistent with the result of another study ^22^ which revealed that the majority of nurses’ age were 35 years old and above. Furthermore, the results showed that the majority (60.9%) of the ICU nurses were female. The high proportion of female nurses could be related to the fact that the profession of nursing remains a female-dominant profession. This finding is similar to a previous study ^16^ that showed the majority (75%) of the participants were female nurses in the context of Egypt.

The current study revealed that more than half of the ICU nurses had a three-year nursing diploma and five years or less of work experience, either as general nurses or as ICU nurses. This could be because studying nursing education is more accessible in many nursing institutes all over Yemen, while there are a limited number of nursing colleges that have opened in the last few years. This finding is nearly in line with a previous study ^16^ that reported most nurses had a diploma with one to four years of working experience. The results also revealed that 59.20% of ICU nurses had never taken any previous training courses on EN and 61.5% of them specified colleges and nursing institutes as the main sources of knowledge about the management of EN for them. This result is consistent to that of Mula ^22^, who found that 72.5% of the participants obtained knowledge about EN during college studying or nursing schools. However, our results contradict the results by Ramuada ^23^ who found that in-service training courses (24.9%) was the most frequent source for knowledge, followed by collages or schools of nursing education (20.6%).

The key finding in the current study was that the ICU nurses exhibited an inadequate level of knowledge concerning EN management. However, only 10.90% of the ICU nurses had an overall adequate level of knowledge regarding the EN management as a whole and the levels of adequate knowledge of it before administration, during administration and after administration were (16.10%, 5.80% and 9.20%, respectively). Such an inadequate knowledge among most Yemeni ICU nurses might be due to the inadequacy of in-service refreshing training’ courses or because hospitals have not a clear and updated guideline protocol that ICU nurses can access and adhere to at work. As mentioned above, 59.20% of ICU nurses had never engaged in training courses about EN, and most had a three-year nursing diploma and had five-years working experience or less, which supports our speculation. Our findings highlighted the gap in ICU nurses’ knowledge regarding the EN management, which indicates the necessity for conducting in-service training courses that focus on EN management. The results are consistent with an earlier study ^10^ which assessed knowledge and practice among 85 nurses working in ICU concerning EN management. The result revealed that ICU nurses had a low and inadequate level of knowledge about EN management. In another similar study ^24^ that assessed nurses’ knowledge and practice regarding use of a nasogastric tube (NGT) in medications administration for ICU patients, an unsatisfactory level of knowledge among nurses was found. Additionally, another study ^25^ assessing nurses’ knowledge level about nutrition revealed that nurses had poor knowledge of nutrition. However, the results of the current study are inconsistent with the result of Al-Hawaly, Ibrahim and Qalawa ^18^ who found that the majority of the respondents had a satisfactory overall level of knowledge concerning NGT nutrition administering. Likewise, the result disagrees with the result of Carlos, Costa and Simino ^26^, who registered a satisfactory level of nurses’ knowledge concerning nutritional therapy.

Another key finding of this study is that the level of ICU nurses’ knowledge regarding EN management was significantly associated with the educational level (χ2 = 13.141, *P*= 0.011). In other words, a high educational level in nursing was associated with a higher level of knowledge. This could be attributed to the fact that faculties of nursing focus more on the theoretical aspect, while health institutes pay considerable attention to the practical aspect. This finding is similar to those of Abdullah *et al*., ^24^, Shahin *et al*., ^10^ and Taha and Said ^16^, who found that the educational level was positively significant associated with the level of the nurses’ knowledge about EN, whereas those who had a bachelor degree achieved a higher level of knowledge compared to those with less educational level. However, our finding was inconsistent with the results of Aml, Manal and Fatamah ^27^, who reported no difference in knowledge level based on the nurses’ educational qualifications. This discrepancy is possibly because of the differences in the curriculum of educational institutions. Furthermore, it was found that a significant association between the knowledge level and the sources of information related to EN management (χ2= 13.948, *P*= 0.030). The result implied that colleges and institutes of nursing represent the main source for respondents' knowledge about EN management. This result could be supported by the result of the present study as the majority (59.2%) of the respondents had never attended training courses related to EN management and only 7.5% of them used other resources for gaining knowledge about EN. Our result is in line with those of Abdullah *et al*. ^24^, who found that doctors and previously working experience were the major sources for the nurses’ knowledge regarding EN management. The result also agrees with that of Morphet *et al*. ^17^ concerning colleagues as the main source of respondents' knowledge. Yet, this disagrees with that result concerning the hospital policies and protocols as the source for the respondents’ knowledge about EN. In contrast, the result is incompatible with that found by Al Kalaldeh, Watson and Hayter ^28^, as the researchers reported that the internet was the major source of knowledge about EN among the other sources for nurses.

On the other hand, there were no significant associations between the level of ICU the gap in ICU nurses’ knowledge regarding EN management and their age, sex, working experience and training courses about EN management. The results are consistent with those of Penland ^29^ in relation to the respondents’ age, as the researcher reported that the respondents’ knowledge level was not significantly associated with age. However, our results are in disagreement with the results of studies by Taha and Said ^16^ and Mooi ^30^ in relation to the nurses’ age and years of working experience, as the researchers found that there was a highly statistically significant association between knowledge level regarding EN management and the respondents' age and previous working experience. This discrepancy might be attributed to the nature of training courses provided and the differences in the respondents' data, where it was found that young and newly graduated nurses had been more receptive, more tolerant and have a more potent memory.

The current study has some limitations that should be addressed in future research. The study was exclusive to assessment of nurses’ knowledge in ICUs at public hospitals in Sana'a, the capital city of Yemen. Thus, the findings should be dealt with caution. Besides, future studies should evaluate nurses’ practices in different hospitals and settings.

## Conclusions

Based on the findings of this study, it can be concluded that Yemeni ICU nurses had an inadequate level of knowledge about EN management. Accordingly, improving such knowledge regarding EN management is extremely needed. To this end, holding an in-service training courses in EN management among ICU Yemeni nurses and conduct such training courses on a regular basis are highly recommended.

## Data availability

### Underlying data

Figshare: ICU nurses’ knowledge about EN. https://doi.org/10.6084/m9.figshare.12542327 ^21^

This file contains the individual-level responses of all participants to each question asked.

### Extended data

Figshare: Questionnaire on ICU nurses’ knowledge regarding EN. https://doi.org/10.6084/m9.figshare.12643988 ^19^.

This file contains an English-language blank copy of the questionnaire used in this study.

Data are available under the terms of the Creative Commons Attribution 4.0 International license (CC-BY 4.0).
