# Supplementary material for: Associated ICU nurses' characteristics to clinical enteral nutrition knowledge at public hospitals in Sana'a, Yemen: A basis for remodeling safety and quality of care standards
Source: F1000Res. 2023 Jul 5;9:759. Originally published 2020 Jul 22. [Version 3] doi: 10.12688/f1000research.25041.3 (PMC10338981; doi:10.12688/f1000research.25041.3)
Supplement: Peer review history

# Peer review: Reviewer response for version 3

**DOI:** 10.5256/f1000research.152499.r184329

**Published:** 2023-07-12

**Authors:** Joannes Paulus Tolentino Hernandez

**Affiliations:** 1 Helene Fuld College of Nursing, New York, NY, USA

Please mention the normality test result for nurses' knowledge under "Results".

 Please edit this sentence for grammar correction/enhancement: "This would enable decision-makers develop a need-based plan to fill the existing gap, and Yemeni nurses' knowledge would allowing them to grow professionally and provide safe health-care services."

**Suggestion:** This would enable decision-makers to develop a need-based plan that can fill the existing gap about Yemeni nurses' knowledge. As an eye-opener study, any contemplated resolution in the forthcoming will be expected to allow Yemeni nurses continual professional growth while underscoring the knowledge progression from current EN training strengths, weaknesses, opportunities, and 'threats' (barriers in clinical practice leadership). Moreover, they will be able to provide and to sustain safe health-care services.

 "Vensim PLE's system dynamics modeling can also be utilized to demonstrate the impact of training on care improvements." -- Please remove "Vensim PLE's" and then change "demonstrate" into visualize.

**Note.** Once the final adjustments above are incorporated, the manuscript can move forward to indexing. Congratulations!

Is the work clearly and accurately presented and does it cite the current literature?

Partly

If applicable, is the statistical analysis and its interpretation appropriate?

Partly

Are all the source data underlying the results available to ensure full reproducibility?

No

Is the study design appropriate and is the work technically sound?

Partly

Are the conclusions drawn adequately supported by the results?

Partly

Are sufficient details of methods and analysis provided to allow replication by others?

Yes

Reviewer Expertise:

Nursing, Communication, Complex Networks, Technology, Robotics

I confirm that I have read this submission and believe that I have an appropriate level of expertise to confirm that it is of an acceptable scientific standard.

# Peer review: Reviewer response for version 2

**DOI:** 10.5256/f1000research.140366.r167311

**Published:** 2023-05-10

**Authors:** Joannes Paulus Tolentino Hernandez

**Affiliations:** 1 Helene Fuld College of Nursing, New York, NY, USA

First of all, reading the manuscript gives vital information of the quality of healthcare that warrants a quality overhaul. I am saddened by the findings although it is critical to make everyone know through research dissemination so readers can ruminate on culturally-relevant training and support for nurses, joint policy-making between implementers and surveillance agencies, and exercise of governance for reviewing the standards for patient safety.

 Second, the descriptive statistics have underscored the reality that met the aims of the study. However, the data distribution could have been indicated and explored for skewness in order to expose the gaps in knowledge distribution. This knowledge distribution may reveal implicit 'disparity' (the access of resources and culturally inflated communicative actions). There is discourse of representation by the data points.

 Third, the Chi-square tests and Fisher exact test did work, but if the authors can consider finding causal relationships then it would be a lot meaningful at the level of designing an intervention that targets specific factors/variables.

 Fourth, establishing that Yemeni nurses have inadequate knowledge should not only be exploratory rather explanatory. Plans for training improvement must be articulated explicitly. It is clear that the authors just wanted to tell the world about a pervasive problem in Yemen, but the problem is at the healthcare system level. So, it may not only be a case of enteral nutrition.

 Fifth, I have these suggestions:

- The title will be better if revised to: "Associated ICU nurses' characteristics to clinical enteral nutrition knowledge in Sana'a, Yemen public hospitals: A basis for remodeling safety and quality of care standards".
- The discussion must connect the level of knowledge with (threat to) patient safety and quality improvements required. SWOT analysis is highly suggested.
- On the other hand, please consider using system dynamics modeling at Vensim PLE. Here is a tutorial. This can be useful to show the impact of training to improvements in care. This serves as an attractive and alternative modeling.
- p-value of less than .05 must be statistically significant.
- This paper should be solution-oriented.

Is the work clearly and accurately presented and does it cite the current literature?

Partly

If applicable, is the statistical analysis and its interpretation appropriate?

Partly

Are all the source data underlying the results available to ensure full reproducibility?

No

Is the study design appropriate and is the work technically sound?

Partly

Are the conclusions drawn adequately supported by the results?

Partly

Are sufficient details of methods and analysis provided to allow replication by others?

Yes

Reviewer Expertise:

Nursing, Communication, Complex Networks, Technology, Robotics

I confirm that I have read this submission and believe that I have an appropriate level of expertise to confirm that it is of an acceptable scientific standard, however I have significant reservations, as outlined above.

# Response

**Published:** 2023-07-01

**Authors:** Gamil Ghaleb Alrubaiee

**Affiliations:** Al-Razi University, Sana'a, Yemen, Yemen

**RESPONSE TO COMMENTS**

 Thank you for your email, and we appreciate all your suggestions for improving the manuscript's quality.

 The responses to the comments are as follows.

**COMMENT # 1:** First of all, reading the manuscript gives vital information of the quality of healthcare that warrants a quality overhaul. I am saddened by the findings although it is critical to make everyone know through research dissemination so readers can ruminate on culturally-relevant training and support for nurses, joint policy-making between implementers and surveillance agencies, and exercise of governance for reviewing the standards for patient safety.

**RESPONSE:** Thank you for your comments. Indeed, our findings were based on the predetermined study objectives. Based on the current findings, we stated that "this would enable decision-makers to develop a need-based plan to fill the existing gap, and Yemeni nurses' knowledge would allow to grow professionally and provide safe health-care services. However, we will consider your valuable comment in our future work.

**COMMENT # 2:** Second, the descriptive statistics have underscored the reality that met the aims of the study. However, the data distribution could have been indicated and explored for skewness in order to expose the gaps in knowledge distribution. This knowledge distribution may reveal implicit 'disparity' (the access of resources and culturally inflated communicative actions). There is discourse of representation by the data points.

**RESPONSE:** Thank you for your comment. We conducted skewness in order to expose the gaps in knowledge distribution. As shown in table and figure, the distribution of knowledge was symmetric.

**COMMENT # 3:** Third, the Chi-square tests and Fisher exact test did work, but if the authors can consider finding causal relationships then it would be a lot meaningful at the level of designing an intervention that targets specific factors/variables.

**RESPONSE:** Thank you for your comment. We did, in fact, perform the Chi-square and Fisher exact tests. Unfortunately, because it was not one of our study aims, we did not explore the causal relationships. However, we have included this useful suggestion in our study recommendations for further research. Please see lines 4-6 in the last paragraph of the discussion section.

**COMMENT # 4: **Fourth, establishing that Yemeni nurses have inadequate knowledge should not only be exploratory rather explanatory. Plans for training improvement must be articulated explicitly. It is clear that the authors just wanted to tell the world about a pervasive problem in Yemen, but the problem is at the healthcare system level. So, it may not only be a case of enteral nutrition.

**RESPONSE:** Thank you for your insightful observation and comment. In fact, our study objective was to explore the level of ICU Nurses' knowledge regarding enteral nutrition. Based on our study findings, we have discussed and explained the findings, in discussion section. Please see the discussion part. Based on your advice, we have already added a recommendation for additional research to examine the causal relationships.

**COMMENT** **# 5: **Fifth, I have these suggestions:

**COMMENT:** The title will be better if revised to: "Associated ICU nurses' characteristics to clinical enteral nutrition knowledge in Sana'a, Yemen public hospitals: A basis for remodeling safety and quality of care standards". **RESPONSE:** Thank you for your comment. We have updated the study title based on your suggestion. **COMMENT:** The discussion must connect the level of knowledge with (threat to) patient safety and quality improvements required. SWOT analysis is highly suggested.

**RESPONSE:** Thank you for your comment. We employed the elements of discussion in this present study. We humbly believe that SWOT analysis in the discussion part can be used in our future interrogations. **COMMENT:** On the other hand, please consider using system dynamics modeling at Vensim PLE. Here is a tutorial. This can be useful to show the impact of training to improvements in care. This serves as an attractive and alternative modeling. **RESPONSE:** Thank you for your comment and useful tutorial link. Indeed, our study was not aimed to show the impact of training to improvements in care. Nevertheless, we will take this into consideration in our future research endeavor and will put this in one of our conclusion and recommendations. Please see lines 5&6 of the conclusion section. **COMMENT:** p-value of less than .05 must be statistically significant.

**RESPONSE:** Thank you for your comment. We would like to clarify that our hypothesis was that the cut point value for significant, “A p-value of ≤0.05 reported as statistically significant” **COMMENT:** This paper should be solution-oriented. **RESPONSE:** Thank you for your comment. Based on the study findings, we proposed a solution to improve the nurses' knowledge regarding enteral nutrition. Please see the conclusion section.

# Peer review: Reviewer response for version 2

**DOI:** 10.5256/f1000research.140366.r154826

**Published:** 2022-11-03

**Authors:** Khaled Mohammed Al-Sayaghi

**Affiliations:** 1 Critical Care and Emergency Nursing, College of Nursing, Taibah University, Medina, Saudi Arabia; 2 Faculty of Medicine and Health Sciences, Sana'a University, Sana'a, Yemen

**Approved**

Is the work clearly and accurately presented and does it cite the current literature?

Yes

If applicable, is the statistical analysis and its interpretation appropriate?

Partly

Are all the source data underlying the results available to ensure full reproducibility?

Yes

Is the study design appropriate and is the work technically sound?

Yes

Are the conclusions drawn adequately supported by the results?

Yes

Are sufficient details of methods and analysis provided to allow replication by others?

Yes

Reviewer Expertise:

Critical Care and Emergency Nursing, Medical surgical Nursing, nutritional support in ICU

I confirm that I have read this submission and believe that I have an appropriate level of expertise to confirm that it is of an acceptable scientific standard.

# Peer review: Reviewer response for version 1

**DOI:** 10.5256/f1000research.27625.r75190

**Published:** 2020-12-10

**Authors:** Hiroyuki Ohbe

**Affiliations:** 1 Department of Clinical Epidemiology and Health Economics, University of Tokyo, Hongo, Bunkyo-ku, Tokyo, Japan

The manuscript "Intensive care nurses' knowledge of enteral nutrition at public hospitals in Sana'a, Yemen: a cross-sectional survey" by Talal Ali Hussein Al-Qalah, *et al. *is a descriptive cross-sectional study from four public hospitals in the capital city of Yemen. The authors intended to assess the level of ICU nurses’ knowledge regarding the management of enteral nutrition. The authors recruit 174 nurses in 2019. The authors found that 46% of them had an inadequate level of knowledge regarding enteral nutrition management. From my impressions, the aim of this study may not be interested in countries other than Yemen.

1. Introduction: Please clearly state “what is unknown” in their topic. It is unclear why the authors did this study.
2. Methods: Sample size determination: I’m not familiar with the equation the authors used. Can the authors provide the reference to support this sample size calculation?
3. Methods: Did the authors use the questionnaire which was previously developed or which was newly created? If the authors use the questionnaire which was newly created, how and why the authors could define that a score of 50% or less was considered inadequate, 51–75% moderate, while 76% and above was considered adequate. The cut-off score should be carefully selected.
4. Methods: Please clearly describe how to perform the multinomial logistic regression. How to obtain the p-values in Table 2? Multinomial logistic regression? Chi-square test?
5. Methods: Because the cells in Table 2 contained under <5, Fisher exact test is more suitable than chi-square test.
6. Discussion: If the authors used the different questionnaire used in the previous study, how and why the authors could compare the results?
7. Conclusions: Is there any evidence that training courses in EN management improve clinical outcomes? Based on the results of your study, the only thing the authors revealed was that low SES was associated with inadequate knowledge. Experience and training courses were not associated with improved knowledge. Therefore, the conclusion should be based on their results.
8. Discussion: What is new in this study? What can the readers act from this paper all over the world?

Is the work clearly and accurately presented and does it cite the current literature?

Partly

If applicable, is the statistical analysis and its interpretation appropriate?

Partly

Are all the source data underlying the results available to ensure full reproducibility?

Partly

Is the study design appropriate and is the work technically sound?

Partly

Are the conclusions drawn adequately supported by the results?

Partly

Are sufficient details of methods and analysis provided to allow replication by others?

Partly

Reviewer Expertise:

Nutrition support in critically ill patients. Clinical epidemiology.

I confirm that I have read this submission and believe that I have an appropriate level of expertise to state that I do not consider it to be of an acceptable scientific standard, for reasons outlined above.

# Response

**Published:** 2022-10-26

**Authors:** Gamil Ghaleb Alrubaiee

**Affiliations:** Al-Razi University, Sana'a, Yemen, Yemen

**COMMENT # 1:** Introduction: Please clearly state “what is unknown” in their topic. It is unclear why the authors did this study.

**RESPONSE:** Thank you for your comment. We agree with your comment that it was unclear in the introduction why the authors conducted the study, but it is stated clearly in the abstract.

 Please refer to lines 2–4 in the background section. In response to your comment, we have clarified in the introduction why the authors conducted this study. Please see paragraph 4, lines 10, 11, and 12 of the introduction.

**COMMENT # 2:** Methods: Sample size determination: I’m not familiar with the equation the authors used. Can the authors provide the reference to support this sample size calculation?

**RESPONSE:** Thank you for your comment. The equation we used to determine sample size in our study was found in many textbooks and cited by many other previous studies. We have included a reference according to your comment. Please see sample size determination section, lines 3.

**COMMENT # 3:** Methods: Did the authors use the questionnaire, which was previously developed, or which was newly created? If the authors use the questionnaire which was newly created, how and why the authors could define that a score of 50% or less was considered inadequate, 51–75% moderate, while 76% and above was considered adequate. The cut-off score should be carefully selected.

**RESPONSE:** Thank you for your comment. We did, in fact, use an adapted questionnaire and didn't develop a new one. The word "developed" is used incorrectly in this statement (A self-administered questionnaire was developed). We have changed the word “developed” by “adapted” to be clear. In addition, we have added cited studies based on Reviewer 2's comment. Please see study instrument section, lines 2.

 The cut-off score used in this study as a scoring system was determined by previous studies rather than by the authors themselves. We already included the reference (Reference #22). Furthermore, many previous studies used a Bloom-based knowledge scoring system, we have added one of these studies. Please see the reference #23.

**COMMENT # 4: **Methods: Please clearly describe how to perform the multinomial logistic regression. How to obtain the p-values in Table 2? Multinomial logistic regression? Chi-square test?

**RESPONSE:** Thank you for your insightful observation and comment. In fact, we did not use multinomial logistic regression in our data analysis; instead, we used the Chi-square test to determine the relationships between ICU nurses' knowledge and the selected socio-demographic variables and obtain p-values. Based on your valuable input and that of Reviewer 2, the phrase "multinomial logistic regression" has been removed, and the analysis statement has been corrected. Please see data analysis section, lines 4.

**COMMENT # 5: **Methods: Because the cells in Table 2 contained under <5, Fisher exact test is more suitable than chi-square test.

**RESPONSE:** Thank you for your comment. We fully agree with you. We use the Fisher exact test to re-analyze the data in this table and re-write the table results accordingly. Please see Table 2. Please also see line 4 for the addition of this test to the data analysis section.

**COMMENT # 6: **Discussion: If the authors used the different questionnaire used in the previous study, how and why the authors could compare the results?

**RESPONSE:** Thank you for your comment. Our questionnaire was not significantly different from those used in previous studies. We simply modified an existing questionnaire rather than creating a new one. As a result, we compared our study findings to those of related studies with similar intended outcomes.

**COMMENT # 7: **Conclusions: Is there any evidence that training courses in EN management improve clinical outcomes? Based on the results of your study, the only thing the authors revealed was that low SES was associated with inadequate knowledge. Experience and training courses were not associated with improved knowledge. Therefore, the conclusion should be based on their results.

**RESPONSE:** Thank you for your comment. Thank you for your comment. Our conclusion is derived from our results and there is no sentence implied that training courses in EN management improve clinical outcomes but improve the awareness and knowledge. this is what we concluded based on our findings and this is supported by many previous studies. Please see our conclusion section.

**COMMENT # 8: **Discussion: What is new in this study? What can the readers act from this paper all over the world?

**RESPONSE:** Thank you for your comment. This study, like many others around the world, has clear objectives. These are stated clearly at the end of the introduction section.

 These are explicitly stated at the end of the introduction. According to the study findings, we believe that the situation regarding Yemeni nurses' enteral nutrition care knowledge becomes clear. This would help decision makers develop a plan to fill this knowledge gap, and as a result, Yemeni nurses' knowledge would grow professionally, and they would be able to provide safe health care services. Furthermore, the findings of this study will serve as a data base for future research and will provide potential readers with information about the current situation of Yemen's nurses. We've added a paragraph to explain the study's implications. Please see the discussion section, paragraph 4, Lines 1-6.

# Peer review: Reviewer response for version 1

**DOI:** 10.5256/f1000research.27625.r67783

**Published:** 2020-08-03

**Authors:** Khaled Mohammed Al-Sayaghi

**Affiliations:** 1 Critical Care and Emergency Nursing, College of Nursing, Taibah University, Medina, Saudi Arabia; 2 Faculty of Medicine and Health Sciences, Sana'a University, Sana'a, Yemen

**Abstract:**

- The results part in the abstract should focus on the main results such as the areas with high level of knowledge and areas with lower level of knowledge. Remove the excessive description of the sample socio-demographic characteristic.
- Conclusion part has been written as a recommendation only. It will be better if you summarize the findings then make a brief recommendation.

**Methods: **

- Under the setting subtitle no need to mention the names of the hospitals. Maintaining the confidentiality of participant hospitals. No need to mention the duration of the study under the setting subtitle, it is mentioned under data collection.
- Delete this ‘According to Al-Hawaly, Ibrahim, and Qalawa’ and write : according to a previous study ^(..)^ ….
- Instead of citing the tool they have developed and used, authors must mention and cite the references and literature that had been used in developing the tool.
- Per authors, the second part of data collection instrument contains three subsections which are the before EN administration, during EN administration, and after EN administration. Accepting that this categorization is correct, it is not clear which items are under each subsection. Moreover, most of the 17 items are not related to the before, during, and after EN management, they are related to general knowledge about the EN (such as the indications, goals, benefits, complications, routs, methods, types and contents of EN formula, …….. etc.), even there is an item asking about the types of nutritional support in general (enteral, parenteral, combination, ..etc.). More suitable grouping and categorizing must be used to organize the instrument or to leave it without any categorization it will be better.
- Validity and reliability: delete the word ‘ reliability’ and replace it by ‘ internal consistency’.
- Under the data analysis subtitle, they mentioned that a multinomial logistic regression test was conducted. In fact, no multinomial logistic regression test was done. So , delete ‘ multinomial logistic regression’.

**Results:**

- In results, the use of some terms (such as most, majority) in describing the results need a revision. For example: 49.40% is not a majority.
- Table 2 presents the frequency and percentage of the participants and compares between the subgroups using the Chi-square tests. I think it will be better if the table presents the knowledge means scores and standard deviations of the subgroups and compare between them by the t or f tests. After that multiple regression can be performed.

**Discussion:**

- Discussion must be on the main important findings based on the objectives of the study. No need to write two paragraphs at the beginning of the discussion about the participants' characteristics such as age, gender, educational levels, and work experience. Describing those characteristic in results part is enough.

**General **

- The paper needs English language editing.

Is the work clearly and accurately presented and does it cite the current literature?

Yes

If applicable, is the statistical analysis and its interpretation appropriate?

Partly

Are all the source data underlying the results available to ensure full reproducibility?

Yes

Is the study design appropriate and is the work technically sound?

Yes

Are the conclusions drawn adequately supported by the results?

Yes

Are sufficient details of methods and analysis provided to allow replication by others?

Yes

Reviewer Expertise:

Critical Care and Emergency Nursing, Medical surgical Nursing, nutritional support in ICU

I confirm that I have read this submission and believe that I have an appropriate level of expertise to confirm that it is of an acceptable scientific standard, however I have significant reservations, as outlined above.

# Response

**Published:** 2022-10-26

**Authors:** Gamil Ghaleb Alrubaiee

**Affiliations:** Al-Razi University, Sana'a, Yemen, Yemen

**COMMENT # 1:** Abstract: The results part in the abstract should focus on the main results such as the areas with high level of knowledge and areas with lower level of knowledge. Remove the excessive description of the sample socio-demographic characteristic.

**RESPONSE:** Thank you for your comment. The results section has been rewritten based on your recommendation. Please see the abstract's results section.

**COMMENT # 2:** Conclusion part has been written as a recommendation only. It will be better if you summarize the findings then make a brief recommendation.

**RESPONSE:** Thank you for your comment. Indeed, the findings were summarized in the results section, and the conclusion section focused on the main result, the knowledge gap, and the recommendations based on that result.

**COMMENT # 3:** Methods: Under the setting subtitle no need to mention the names of the hospitals. Maintaining the confidentiality of participant hospitals. No need to mention the duration of the study under the setting subtitle, it is mentioned under data collection.

**RESPONSE:** Thank you for your comment. We have followed your recommendations.

**COMMENT # 4:** Delete this ‘According to Al-Hawaly, Ibrahim, and Qalawa’ and write : according to a previous study(..)

**RESPONSE:** Thank you for your comment. We have followed your recommendations.

**COMMENT # 5:** Instead of citing the tool they have developed and used, authors must mention and cite the references and literature that had been used in developing the tool.

**RESPONSE:** Thank you for your comment. The references for adapted tool were cited as recommended.

**COMMENT # 6:** Per authors, the second part of data collection instrument contains three subsections which are the before EN administration, during EN administration, and after EN administration. Accepting that this categorization is correct, it is not clear which items are under each subsection. Moreover, most of the 17 items are not related to the before, during, and after EN management, they are related to general knowledge about the EN (such as the indications, goals, benefits, complications, routs, methods, types and contents of EN formula, ........ etc.), even there is an item asking about the types of nutritional support in general (enteral, parenteral, combination, ..etc.). More suitable grouping and categorizing must be used to organize the instrument or to leave it without any categorization it will be better.

 Instead of citing the tool they have developed and used, authors must mention and cite the references and literature that had been used in developing the tool.

**RESPONSE:** Thank you for your comment. The items under each subsection were mentioned to be clear for the potential readers. We also replace the word "management" by "care" to be consistent with the general knowledge about the care of EN.

**COMMENT # 7:** Validity and reliability: delete the word ‘reliability’ and replace it by ‘internal consistency’.

**RESPONSE:** Thank you for your comment. We removed the word "reliability" and replaced it with "internal consistency."

**COMMENT # 8:** Under the data analysis subtitle, they mentioned that a multinomial logistic regression test was conducted. In fact, no multinomial logistic regression test was done. So, delete ‘multinomial logistic regression’.

**RESPONSE:** Thank you for your comment. We apologize for mistyping a multinomial logistic regression test. We have removed the term "multinomial logistic regression".

**COMMENT # 9:** Results: In results, the use of some terms (such as most, majority) in describing the results need a revision. For example: 49.40% is not a majority.

**RESPONSE:** Thank you for the comment. We have corrected some terms as recommended.

**COMMENT # 10:** Table 2 presents the frequency and percentage of the participants and compares between the subgroups using the Chi-square tests. I think it will be better if the table presents the knowledge means scores and standard deviations of the subgroups and compare between them by the t or f tests. After that multiple regression can be performed.

**RESPONSE:** Thank you for your comment. Following the recommendation of Reviewer 1, we re-analyze the data in Table 2 using the Fisher exact test rather than the Chi-square test.

 Please see Table 2.

**COMMENT # 11:** Discussion: Discussion must be on the main important findings based on the objectives of the study. No need to write two paragraphs at the beginning of the discussion about the participants' characteristics such as age, gender, educational levels, and work experience. Describing those characteristic in results part is enough.

**RESPONSE: **Thank you for your comment. Based on your recommendation, the two paragraphs about socio-demographic characteristics were removed from the discussion section.

**COMMENT # 12:** **General: **The paper needs English language editing.

**RESPONSE: **Thank you for your comment. The manuscript was reviewed and edited as your recommendation.
